# Evolution and thermodynamics of the slow unfolding of hyperstable monomeric proteins

**DOI:** 10.1186/1471-2148-10-207

**Published:** 2010-07-09

**Authors:** Jun Okada, Tomohiro Okamoto, Atsushi Mukaiyama, Takashi Tadokoro, Dong-Ju You, Hyongi Chon, Yuichi Koga, Kazufumi Takano, Shigenori Kanaya

**Affiliations:** 1 Department of Material and Life Science, Osaka University, 2-1 Yamadaoka, Suita, Osaka 565-0871, Japan; 2 CREST, JST, 2-1 Yamadaoka, Suita, Osaka 565-0871, Japan

## Abstract

**Background:**

The unfolding speed of some hyperthermophilic proteins is dramatically lower than that of their mesostable homologs. Ribonuclease HII from the hyperthermophilic archaeon *Thermococcus kodakaraensis *(Tk-RNase HII) is stabilized by its remarkably slow unfolding rate, whereas RNase HI from the thermophilic bacterium *Thermus thermophilus *(Tt-RNase HI) unfolds rapidly, comparable with to that of RNase HI from *Escherichia coli *(Ec-RNase HI).

**Results:**

To clarify whether the difference in the unfolding rate is due to differences in the types of RNase H or differences in proteins from archaea and bacteria, we examined the equilibrium stability and unfolding reaction of RNases HII from the hyperthermophilic bacteria *Thermotoga maritima *(Tm-RNase HII) and *Aquifex aeolicus *(Aa-RNase HII) and RNase HI from the hyperthermophilic archaeon *Sulfolobus tokodaii *(Sto-RNase HI). These proteins from hyperthermophiles are more stable than Ec-RNase HI over all the temperature ranges examined. The observed unfolding speeds of all hyperstable proteins at the different denaturant concentrations studied are much lower than those of Ec-RNase HI, which is in accordance with the familiar slow unfolding of hyperstable proteins. However, the unfolding rate constants of these RNases H in water are dispersed, and the unfolding rate constant of thermophilic archaeal proteins is lower than that of thermophilic bacterial proteins.

**Conclusions:**

These results suggest that the nature of slow unfolding of thermophilic proteins is determined by the evolutionary history of the organisms involved. The unfolding rate constants in water are related to the amount of buried hydrophobic residues in the tertiary structure.

## Background

Proteins from thermophiles and hyperthermophiles generally exhibit higher stability than their mesostable counterparts [1-4]. Recent research in this field has indicated that some proteins from hyperthermophiles are stabilized by their remarkably slow unfolding rates [5-17]. At present, the molecular mechanism of slow unfolding is not completely clear. Recently, using hydrophobic mutant proteins, Dong *et al*. [18] demonstrated that the hydrophobic effect is one of the reasons for the slow unfolding of ribonuclease HII from the hyperthermophilic archaeon *Thermococcus kodakaraensis *(Tk-RNase HII). However, the attributes of proteins that exhibit unusually slow unfolding are unclear. For example, Tk-RNase HII is kinetically robust [19], whereas RNase HI from the thermophilic bacterium *Thermus thermophilus *(Tt-RNase HI), which has equivalent thermostability but no sequence similarity to Tk-RNase HII, unfolds rapidly. Moreover, the unfolding rate of Tt-RNase HI is comparable with to that of RNase HI from *Escherichia coli *(Ec-RNase HI) [20,21]. The reason for the difference in the unfolding rate is unknown.

Both equilibrium stability and kinetic unfolding/refolding experiments can improve our understanding of the nature of hyperstable proteins. Luke *et al*. [22] listed the hyperthermophilic proteins for which kinetic folding/unfolding parameters have been reported. These are generally oligomers or monomers that show irreversible unfolding. Monomeric proteins with reversible unfolding are suitable for detailed analysis of the essentials of slow unfolding. Only RNase H, cold shock protein (Csp), and ribosomal protein S16 (S16) satisfy these prerequisites [23]. Csp from the hyperthermophilic bacterium *Thermotoga maritima *(Tm-Csp) and ribosomal protein S16 from the hyperthermophilic bacterium *Aquifex aeolicus *(Aa-S16) do not exhibit extremely slow unfolding [24-27]. Data for the unfolding kinetics of RNase H, Csp, and S16 suggest that differences in the type of protein, i.e., whether it is RNase HI or RNase HII, or differences in the organism kingdom, i.e., whether the host organism to which the protein belongs originates from archaea or bacteria, may be the reason for the differences in the unfolding reactions of Tk-RNase HII and Tt-RNase HI.

Based on their sequence similarity, RNases H are classified into two major types--Type 1 RNase H and Type 2 RNase H [28,29]. RNase HI is a member of Type 1 RNase H, whereas RNase HII is a member of Type 2 RNase H. Comparison of the crystal structures of Ec-RNase HI, Tt-RNase HI, and Tk-RNase HII indicates that these enzymes share a folding motif referred to as the RNase H-fold due to its characterization in Ec-RNase HI [30], although there is no sequence similarity between RNase HI and RNase HII. The stability and folding of Ec-RNase HI, Tt-RNase HI, and Tk-RNase HII have been well studied [18-21,31-40].

Archaea and bacteria have divided at an early stage of evolution [41]. Hyperthermophiles and thermophiles in archaea originated in a hot environment but those in bacteria recolonized at a later stage under extreme conditions. The differences in the unfolding characteristics of hyperstable proteins may be ascribed to such differences in the evolutionary history of thermophilic archaea and bacteria.

In this study, we focused on Tm-RNase HII, Aa-RNase HII, and Sto-RNase HI (*Sulfolobus tokodaii*) and compared these proteins with Tk-RNase HII, Tt-RNase HI, and Ec-RNase HI in terms of their stability and unfolding kinetics [19-21,31]. Tm-RNase HII and Aa-RNase HII are proteins from hyperthermophilic bacteria and are Type 2 RNase H proteins. Sto-RNase HI is a protein from a hyperthermophilic archaeon and is a Type 1 RNase H. All these proteins are monomeric. Table 1 summarizes the types and origins of RNases H studied in this paper. The crystal structures of Ec-RNase HI, Tt-RNase HI, Sto-RNase HI, Tk-RNase HII, and Tm-RNase HII have already been determined (PDB ID 2RN2, 1RIL, 2EHG, 1IO2, and 2ETJ) [42-45] (see additional file 1). We used circular dichroism (CD) to study the equilibrium stability and kinetic aspects of guanidine hydrochloride (GdnHCl)-induced unfolding of Tm-RNase HII, Aa-RNase HII, and Sto-RNase HI. These proteins exhibited reversible unfolding during GdnHCl-induced unfolding. The unfolding of these hyperthermophilic origin RNases H induced by GdnHCl concentration jumps is apparently quite slow and similar to that of several hyperthermophilic proteins that exhibit irreversible unfolding. However, the unfolding rate constant in water depends on the kingdom (archaea or bacteria) to which the host microorganisms belong. Based on the results obtained, we discuss the origin of slow unfolding in relation to the attributes of the proteins.

**Table 1:** Types and origins of RNases H.

	Archaea	Bacteria
Type 1 RNase HI	Sto; *Sulfolobus tokodaii* (hyperthermophile)^a^	Ec; *Escherichia coli* (mesophile)
		Tt; *Thermus thermophilus* (thermophile)
Type 2 RNase HII	Tk; *Thermococcus kodakaraensis* (hyperthermophile)	Aa; *Aquifex aeolicus* (hyperthermophile)^a^
		Tm; *Thermotoga maritima* (hyperthermophile)^a^

^a ^This work.

## Results

### CD spectra and reversible unfolding in GdnHCl

The far-UV spectra of Tm-RNase HII, Aa-RNase HII, and Sto-RNase HI were recorded in the absence and presence of GdnHCl at 25°C (see additional file 2). The spectra represent the proteins in their native and GdnHCl denatured states in the absence and presence of GdnHCl. The spectra of the folded condition obtained from the denatured state by dilution of GdnHCl were also recorded. The results demonstrate that GdnHCl-induced unfolding of these proteins is almost completely reversible under each condition (see additional file 2). Experiments on GdnHCl-induced unfolding of these proteins were carried out under conditions of reversible unfolding (see below). It has been shown that Tk-RNase HII, Tt-RNase HI, and Ec-RNase HI undergo reversible unfolding in the presence of denaturants [19,20,31]. RNases H are considered to be good models for clarifying the mechanisms of conformational stability and folding of hyperstable proteins. RNase HI from the psychotrophic bacterium *Shewanella onedensis *MR-1 was also shown to unfold reversibly in the presence of chemical denaturants [46-48].

### Equilibrium stability of GdnHCl-induced unfolding and stability profile

**Figure 1:**
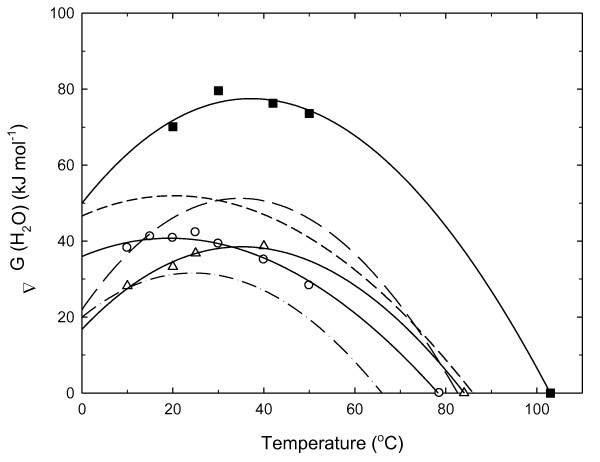
**Thermodynamic stability profiles of RNases H**. Open circles represent the temperature dependence of ΔG(H_2_O) of Tm-RNase HII at pH 7.5; open triangles, those of Aa-RNase HII at pH 5.0; and closed squares, those of Sto-RNase HI at pH 3.0. The T_m _at which ΔG(H_2_O) becomes zero were estimated from the heat-induced unfolding experiments. The lines represent the fit of Eq. (3). Long-dashed line represents the stability profiles of Tk-RNase HII; the short-dashed line, that of Tt-RNase HI; and the one-point dashed line, that of Ec-RNase HI [19,20].

The equilibrium stabilities of Tm-RNase HII, Aa-RNase HII, and Sto-RNase HI were evaluated by GdnHCl-induced denaturation, and the changes were monitored by CD at various temperatures (see additional file 3). Reactions reached equilibrium within 3 days for Tm-RNase HII and Aa-RNase HII and within 3 weeks for Sto-RNase HI, suggesting that the unfolding of these proteins was slow. These proteins exhibit a two-state transition. The ΔG(H_2_O) value at each temperature was calculated using Eqs. (1) and (2). The resultant ΔG(H_2_O) values were plotted as a function of the temperature (stability profile) in Figure 1 and were fitted to Eq. (3). When fitting these values to Eq. (3), the T_m _value, which is the thermal denaturation temperature obtained from the heat-induced unfolding experiment (see below), was used (ΔG(H_2_O) = 0 at T_m_). Figure 1 shows the data for Tk-RNase HII, Tt-RNase HI, and Ec-RNase HI. The values of T_m_, ΔC_p_, ΔH_m_, T_s_, and ΔG(T_s_) of RNases H are summarized in Table 2. ΔC_p _represents the difference in the heat capacity of the native and unfolded states; ΔH_m _is the enthalpy change of the unfolding at T_m_; T_s _is the temperature at which the protein exhibits the maximal ΔG(H_2_O) value; and ΔG(T_s_) is the ΔG(H_2_O) value at T_s_.

**Table 2:** Comparison of the thermodynamic parameters of Tm-, Aa-, Tk-RNases HII and Sto-, Tt-, Ec-RNases HI.

	RNase HII			RNase HI		
	Tm	Aa	Tk^a^	Sto	Tt^b^	Ec^b^
T_m _(°C)	78.6	84.0	82.8	102.0	86.0	66.0
ΔC_p_ (kJ mol^-1 ^K^-1^)	7.3 ± 0.6	10.4 ± 0.6	14.5 ± 1.8	11.9 ± 1.2	7.5 ± 0.4	11.3 ± 0.8
ΔH_m_ (kJ mol^-1^)	471 ± 19	549 ± 21	745 ± 49	858 ± 45	548 ± 21	502 ± 17
T_s _(°C)	19.3	35.2	40.0	37.4	20.0	24.0
ΔG(T_s_) (kJ mol^-1^)	40.7	38.5	51.2	77.5	53.1	31.3

^a ^Data from ref. [19].

^b ^Data from ref. [20].

RNases H from thermophiles and hyperthermophiles are more stable than Ec-RNase HI over most of the temperature ranges examined. In particular, Sto-RNase HI has much higher stability. However, the thermodynamic parameters and stabilization mechanism from the viewpoint of equilibrium stability for the hyperstable RNases H differ depending on the proteins (see Discussion below).

### Thermal stability of heat-induced unfolding

Heat-induced unfolding of Sto-RNase HI has already been analyzed by differential scanning calorimetry (DSC) [44]. The T_m _of Sto-RNase HI is 102.0°C. The thermal denaturation curves of Tm-RNase HII and Aa-RNase HII were recorded by measuring the change in CD. The thermal denaturation of these proteins was almost completely reversible in the presence of various concentrations of GdnHCl (see Methods). T_m _was calculated by curve fitting the resultant CD spectrum versus temperature based on a least-squares analysis using Eq. (4). The T_m _values of Tm-RNase HII and Aa-RNase HII in the absence of GdnHCl were estimated to be 78.6°C and 84.0°C, respectively, and were comparable to those of Tt-RNase HII (86.0°C) and Tk-RNase HII (82.8°C) [19,20].

### Kinetics of GdnHCl-induced unfolding

**Figure 2:**
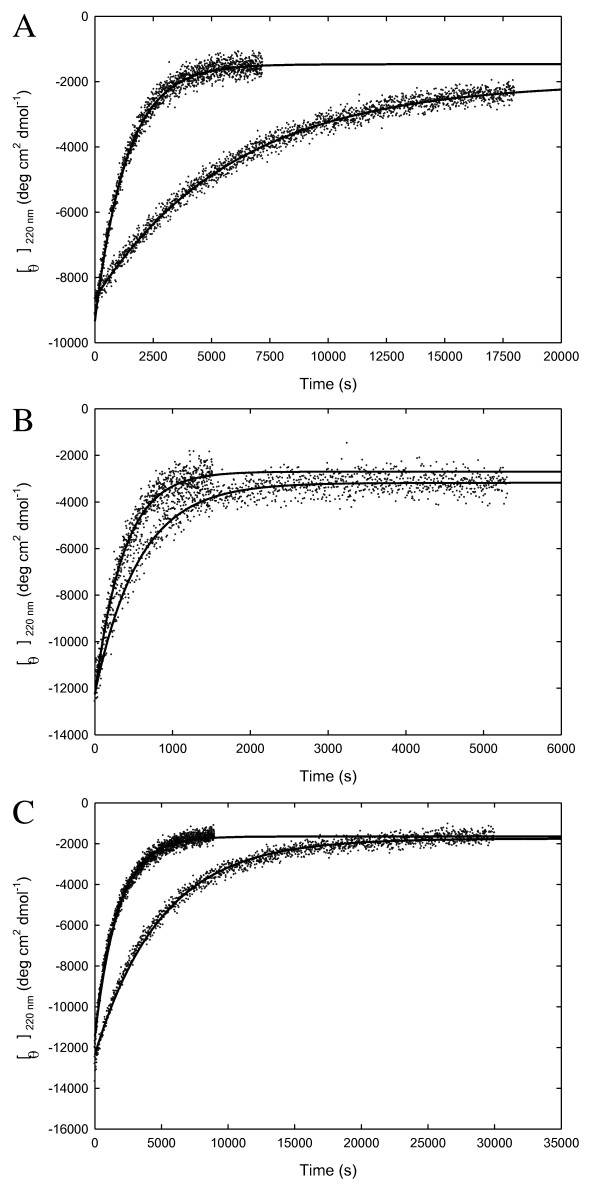
**Unfolding kinetic curves of RNases H at 25°C**. (A) Curves 1 and 2 represent the unfolding traces to a final concentration of 4.8 and 3.8 M GdnHCl of Tm-RNase HII at pH 7.5. (B) Curves 1 and 2 represent the unfolding traces to a final concentration of 4.0 and 3.5 M GdnHCl of Aa-RNase HII at pH 5.0. (C) Curves 1 and 2 represent the unfolding traces to a final concentration of 7.5 and 7.0 M GdnHCl of Sto-RNase HI at pH 3.0. The lines represent the fit of Eq. (5).

**Figure 3:**
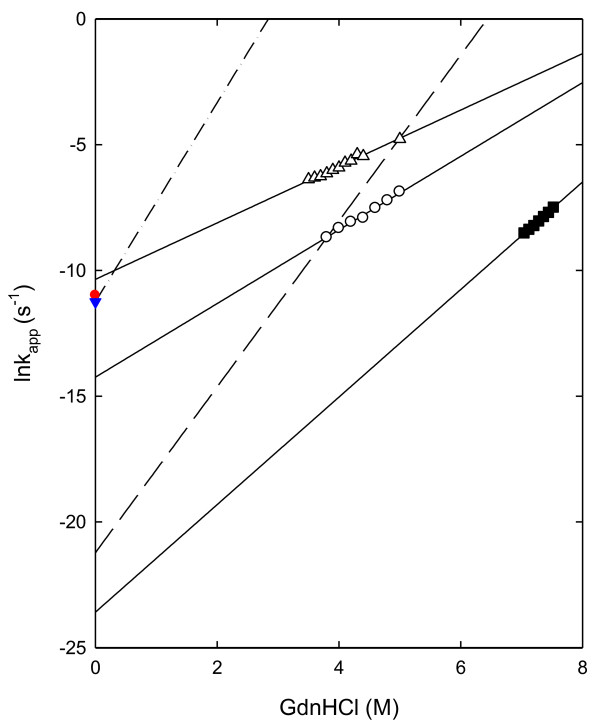
**GdnHCl concentration dependence of the apparent rate constant (ln k_app_) of the unfolding of RNases H at 25°C**. Open circles represent the data of Tm-RNase HII at pH 7.5; open triangles, that of Aa-RNase HII at pH 5.0; and closed squares, that of Sto-RNase HI at pH 3.0. The lines represent the fit of Eq. (6). Long dashed line represent the data of Tk-RNase HII and one-point dashed line represent Ec-RNase HI [19,31]. The red circle and blue triangles represents the k_u_(H_2_O) value obtained from urea-induced unfolding experiments with Ec-RNase HI [33] and Tt-RNase HI [21], respectively.

The kinetics of GdnHCl-induced unfolding of Tm-RNase HII, Aa-RNase HII, and Sto-RNase HI were measured at 25°C. The reaction was initiated by jumps to various GdnHCl concentrations followed by CD measurements. The representative curves were plotted, as shown in Figure 2. All kinetic traces are described well by a single exponential. Figure 3 shows the GdnHCl concentration dependence of the logarithms of the apparent rate constant (k_app_) for the unfolding of RNases H. The logarithms of k_app _linearly increase with an increase in the GdnHCl concentration. Figure 3 also shows the GdnHCl-induced unfolding data for Tk-RNase HII and Ec-RNase HI. The k_app _values for RNases H from hyperthermophiles were significantly lower than those of Ec-RNase HI at all the GdnHCl concentrations examined. By fitting the data to Eq. (6), k_u_(H_2_O), which is the rate constant for unfolding in the absence of GdnHCl, and m_u_, which is the slope of the linear correlation of ln k_u _with the GdnHCl concentration, were calculated. These values are listed in Table 3. Figure 3 shows the urea-induced unfolding data for Ec-RNase HI and Tt-RNase HI [21,33]. The k_u_(H_2_O) values for RNases H are dispersed over six orders of magnitude. The k_u_(H_2_O) value of Aa-RNase HII is similar to that of Ec-RNase HI, whereas the unfolding of Tk-RNase HII and Sto-RNase HI is remarkably slow.

**Table 3:** Comparison of the kinetic parameters of Tm-, Aa-, Tk-RNases HII and Sto-, Tt-, Ec-RNases HI at 25°C.

	RNase HII			RNase HI		
	Tm	Aa	Tk^a^	Sto	Tt	Ec^b^
k_u_(H_2_O) (s^-1^)	6.5 × 10^-7^	3.7 × 10^-5^	6.0 × 10^-10^	5.7 × 10^-11^	- (1.3 × 10^-5^)^c^	1.1 × 10^-5^ (1.7 × 10^-5^)^d^
m_u_ (M^-1 ^s^-1^)	1.46	1.08	3.29	2.14	-	3.66

^a ^Data from ref. [19].

^b ^Data from ref. [31].

^c ^Data from ref. [21]. The k_u_(H_2_O) value was obtained from the urea-induced unfolding experiments.

^d ^Data from ref. [33]. The k_u_(H_2_O) value was obtained from the urea-induced unfolding experiments.

## Discussion

Proteins from hyperthermophiles often exhibit slower unfolding than those from organisms that grow at moderate temperatures [5-17]. However, the extent of the decrease in the unfolding rate of hyperthermophilic proteins relative to that of their mesophilic counterparts varies, depending on the type of protein involved. The attributes of proteins that exhibit unusually slow unfolding have not been elucidated. In this study, we attempted to characterize super slow unfolding and elucidate its mechanism by examining several RNase H proteins from thermophiles and hyperthermophiles.

### Equilibrium stability of hyperstable RNases H

The stability of proteins in solution is quantitatively evaluated by the Gibbs energy changes (ΔG) observed upon unfolding when the reaction is reversible under experimental conditions. The temperature dependence of ΔG (stability profile) provides some information on the thermodynamic stability of proteins and can be expressed by Eq. (3).

Three thermodynamic models have been proposed to explain the high stability of thermostable proteins [49]. In the first model, the entire stability curve is raised to a higher ΔG; in the second, the stability curve is flattened; and in the third, the curve is shifted to a higher temperature. Adherence to all three models is observed in nature, and in many cases, proteins from hyperthermophiles utilize various combinations of these mechanisms [50-55]. The outcome depends on the model used to adapt the proteins to a high temperature.

RNases H from thermophiles and hyperthermophiles are more stable than Ec-RNase HI over most of the temperature ranges examined (Figure 1). In the case of hyperstable RNases HI, Tt-RNase HI adopts the first and second models, and Sto-RNase HI uses the first and third models (Figure 1 and Table 2). There is no data on mesophilic RNase HII. Among the hyperthermophilic RNases HII, Tm-RNase HII has a flatter curve than Aa-RNase HII and Tk-RNase HII as a result of its lower ΔC_p_. The T_s _value of Tm-RNase HII is also lower than that of Aa-RNase HII and Tk-RNase HII. However, ΔG(T_s_) of Tk-RNase HII is higher than that of Tm-RNase HII and Aa-RNase HII. These results suggest that the thermodynamic stabilization of RNases H from thermophiles and hyperthermophiles is achieved through different mechanisms.

### Unfolding of hyperstable RNases H induced by GdnHCl concentration jump

Globular proteins usually unfold in the presence of GdnHCl. Unfolding takes less than 1 min in most small globular proteins. To measure such fast unfolding rates, a stopped-flow instrument is required. Ec-RNase HI is unfolded within 10 s by a final concentration of 3.0 M GdnHCl [31]. In contrast, the unfolding of some hyperstable proteins may take a few hours to several days. Pyrrolidone carboxyl peptidase from the hyperthermophilic archaeon *Pyrococcus furiosus *(Pf-PCP) is unfolded over a period of 1 day or more by a final concentration of 7.7 M GdnHCl [6], and the unfolding of Tk-RNase HII by a final concentration of 3.9 M GdnHCl requires 2 h [19]. The observed unfolding of all hyperstable proteins examined here is much slower than that of Ec-RNase HI in the presence of GdnHCl (Figures 2 and 3). For example, Tm-RNase HII is unfolded by 4.8 M GdnHCl within approximately 2 h; Aa-RNase HII is unfolded by 3.5 M GdnHCl within 1 h; and Sto-RNase HI is unfolded by 7.0 M GdnHCl within about 8 h. Our results suggest that such super slow unfolding in the presence of a chemical denaturant might be a common strategy for achieving higher stability and could be related to the adaptation of hyperthermophilic RNases H to higher temperatures.

### Estimation of the unfolding speed of hyperstable RNases H at zero denaturant concentration

All the RNases H from the hyperthermophiles examined showed a remarkably slow rate of unfolding in the presence of GdnHCl, as described above. However, the dependencies of the apparent rate constants of the unfolding reactions on the GdnHCl concentration varied according to the type of protein (Figure 3). By extrapolating the unfolding rate constants to zero, the denaturant concentration yields the semilogarithmic unfolding rate constants in water (ln k_u_(H_2_O)). It has been reported that k_u_(H_2_O) at 25°C is 1.1 × 10^-5 ^s^-1 ^for GdnHCl-induced unfolding and 1.7 × 10^-5 ^s^-1 ^for urea-induced unfolding of Ec-RNase HI. Furthermore, the values are 1.3 × 10^-5 ^s^-1 ^for urea-induced unfolding of Tt-RNase HI and 6.0 × 10^-10 ^s^-1 ^for GdnHCl-induced unfolding of Tk-RNase HII [19,21,31,33]. These values indicate that Tt-RNase HI unfolds in water on a time scale similar to that of Ec-RNase HI; however, the unfolding rate of Tk-RNase HII is dramatically lower than that of Ec-RNase HI. Therefore, the unfolding of Tk-RNase HII is much slower than that of Tt-RNase HI, although the equilibrium stabilities of Tt-RNase HI and Tk-RNase HII are comparable. Furthermore, although there are no unfolding kinetics data for Ec-RNase HII, it is possible that Ec-RNase HII may not exhibit slow unfolding because Ec-RNase HII is less stable than Ec-RNase HI [56].

The k_u_(H_2_O) value for GdnHCl-induced unfolding at 25°C is 6.5 × 10^-7 ^s^-1 ^for Tm-RNase HII, 3.7 × 10^-5 ^s^-1 ^for Aa-RNase HII, and 5.7 × 10^-11 ^s^-1 ^for Sto-RNase HI (Table 3). The results indicate that Aa-RNase HII unfolds in water on a time scale similar to that of Ec-RNase HI and Tt-RNase HI. In contrast, Sto-RNase HI has remarkably slow unfolding characteristics, as in the case of Tk-RNase HII. The unfolding of Tm-RNase HII is slower than that of Ec-RNase HI, but the difference is much less than that between Ec-RNase HI and Sto-RNase HI. Thus, Tm-RNase HII does not show the extremely slow unfolding reactions.

For Tk-RNase HII and Sto-RNase HI, which exhibit slow unfolding, the experimental conditions were pH 9.0 [19] and pH 3.0, respectively, because the proteins unfold reversibly at each of these pH conditions. At neutral pH where these proteins do not show completely reversible unfolding, the k_u_(H_2_O) value due to GdnHCl-induced unfolding at 25°C was not very different from the value obtained under reversible conditions. For example, the k_u_(H_2_O) value was 9.3 × 10^-10 ^s^-1 ^for Tk-RNase HII at pH 7.5 and 3.3 × 10^-11 ^s^-1 ^for Sto-RNase HI at pH 5.5. Therefore, Tk-RNase HII and Sto-RNase HI unfold extremely slowly at neutral pH.

It should be noted here that the differences in the unfolding rates of hyperstable RNases H reflect the organism kingdom from which the host microorganisms of RNases H originate, i.e., archaea or bacteria. RNases H from thermophilic bacteria, such as Tt-RNase HI, Aa-RNase HII, and Tm-RNase HII unfold faster than similar proteins from thermophilic archaea, Sto-RNase HI, and Tk-RNase HII. This finding is supported by data from other monomeric proteins with reversible unfolding. Tm-Csp and Aa-S16 from hyperthermophilic bacteria do not exhibit extremely slow unfolding [24-27].

### Origin of super slow unfolding

As described above, the nature of slow unfolding depends on the evolutionary history of the organisms. What is the difference between thermophilic archaeal and bacterial proteins? Berezovsky & Shakhnovich [57] reported that proteins from archaea that originated in a hot environment are more compact and hydrophobic than their mesophilic homologs. In contrast, proteins from some bacteria, such as Tm, that recolonized at a later stage under extreme conditions are stabilized by specific interactions such as salt bridges. Mizuguchi *et al*. [58] have shown differences in the amino acid composition of archaeal and bacterial proteins. They concluded that thermal adaptation is achieved in different ways in the archaeal and bacterial kingdoms.

**Figure 4:**
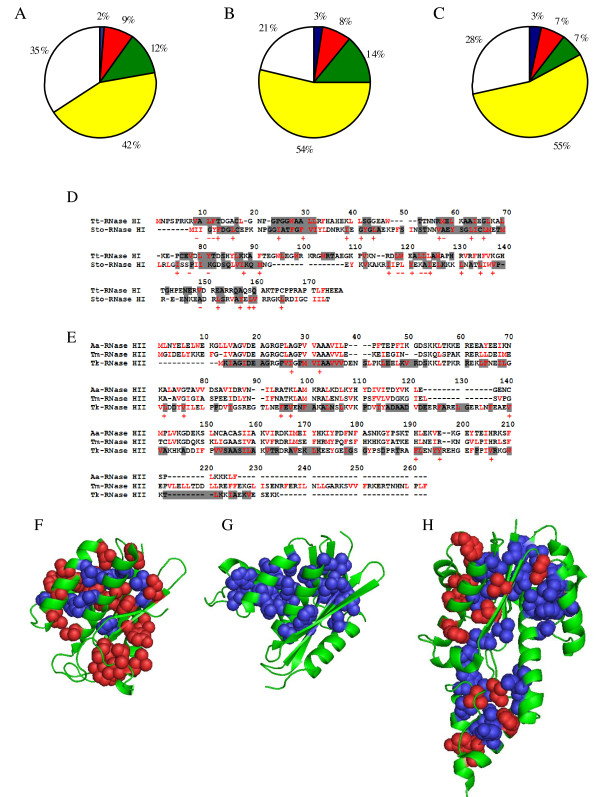
**Characteristics of RNases H**. (A-C) Pie diagrams representing the fraction of hydrophobic, polar, and charged residues in the interior of the tertiary structure of hyperstable RNases H. (A) Tt-RNase HI. (B) Sto-RNase HI. (C) Tk-RNase HII. Blue denotes positively charged residues (Arg and Lys). Red denotes negatively charged residues (Asp and Glu). Green denotes polar residues (Asn, Gln, Ser, and Thr). Yellow denotes hydrophobic residues (Ile, Leu, Met, Phe, Trp, Tyr, and Val). White denotes other residues (Ala, Cys, Gly, His, and Pro). Amino-acid residues with relative solvent accessibility greater than 25% were regarded as residues exposed to solvent [67]. (D and E) Amino acid sequence alignments of hyperstable RNases H. (D) RNases HI. (E) RNases HII. Buried residues (Tt, Sto and Tk) and hydrophobic residues are shown in gray and red, respectively. Crosses indicate buried hydrophobic residues in Sto-RNase HI and Tk-RNase HII with non-buried or non-hydrophobic counterpart in Tt-RNase HI and Aa/Tm-RNases HII, respectively. Minuses indicate buried hydrophobic residues in Tt-RNase HI with non-buried or non-hydrophobic counterpart in Sto-RNase HI. (F-H) Crystal structures of hyperstable RNases H. (F) Tt-RNase HI. (G) Sto-RNase HI. (H) Tk-RNase HII. Buried hydrophobic side-chains (relative solvent accessibility less than 25%) are represented by sphere (blue and red). The buried hydrophobic residues in Sto-RNase HI and Tk-RNase HII with nonburied or nonhydrophobic counterpart in Tt-RNase HI and Aa/Tm-RNases HII are shown in red. The figures were created by PyMOL [68].

The pie diagrams shown in Figures 4A, 4B, and 4C depict the fraction of hydrophobic, polar, and charged residues in the interior of the tertiary structure of hyperstable RNases H. Because the structure of Aa-RNase HII has not been solved and some regions in the crystal structure of Tm-RNase HII have not been determined, we used the structures of Tt-RNase HI, Sto-RNase HI, and Tk-RNase HII. The results reveal that Sto-RNase HI and Tk-RNase HII from archaea are richer in hydrophobic residues than Tt-RNase HI from bacteria. This indicates that super slow unfolding in water is related to the amount of buried hydrophobic residues. The strong effect of hydrophobic interactions on the slow unfolding of Tk-RNase HII supports this hypothesis [18]. We also calculated the fractions of buried amino acid residues of other hyperstable RNase H homologs, the crystal structures of which have been determined. Similar to Sto-RNase HI and Tk-RNase HII, RNases HII from the hyperthermophilic archaea *Pyrococcus furiosus *(1UAX) and *Archaeoglobus fulgidus *(1I39) have more buried hydrophobic residues than Tt-RNase HII (see additional file 4). Furthermore, phylogenetic trees based on thermostable RNases HI and HII clearly show the difference in the characteristics of archaea and bacteria proteins (see additional file 5). These results suggest that archaeal RNases H are stabilized by super slow unfolding but bacterial RNases H are not.

We also calculated the buried area of the polar (O and N) and nonpolar (C and S) atoms in the interior of the proteins because the buried area contributes to protein stability [59]. The buried areas of nonpolar atoms in Tt-RNase HI, Sto-RNase HI, and Tk-RNase HII were 62.6%, 67.3%, and 64.5%, respectively (see additional file 6). Surprisingly, there was no significant difference between bacterial and archaeal proteins, although the hydrophobicity of archaeal RNases H (Sto-RNase HI and Tk-RNase HII) was higher than that of bacterial RNase H (Tt-RNase HI). Therefore, we suggest that the overall hydrophobicity, calculated from the buried area of atoms, does not differ greatly between bacterial and archaeal proteins and contributes to their equilibrium stability. The equlibrium stability of Sto-RNase HI, which is the most hydrophobic RNase H, is considerably higher than that of Tt-RNase HI and Tk-RNase HII (Figure 1). It has been reported that 100 Å^2 ^of buried nonpolar atoms contributes to an equilibrium stability of about 15 kJ mol^-1 ^[59]. In contrast, the estimate from the sequence/structure data (the fractions of amino acid residues in the interior of proteins) differs between bacterial and archaeal proteins [57,58], as described above. This suggests that the hydrophobic interaction arising from the partially exposed hydrophobic side chains may affect the unfolding rate (see below).

Figures 4D and 4E represent the amino acid sequence alignments, buried residues (Tt, Sto and Tk), and hydrophobic residues of thermophilic RNases H. In RNases HI, Sto-RNase HI has 24 buried hydrophobic residues with nonburied or nonhydrophobic counterparts in Tt-RNase HI, whereas Tt-RNase HI has 11 such residues. Tk-RNase HII also possesses 10 buried hydrophobic residues with nonhydrophobic counterparts in Aa-RNase HII and Tm-RNase HII. These residues in Sto-RNase HI and Tk-RNase HII may be possible causes of the slow unfolding rate. These results will be confirmed in the near future by amino acid substitution experiments. Figures 4F, 4G, and 4H show the crystal structures of the buried hydrophobic residues of Tt-RNase HI, Sto-RNase HI, and Tk-RNase HII. Among RNases HI, Sto-RNase HI has more buried hydrophobic residues than Tt-RNase HI. Furthermore, it seems that the the 24 buried hydrophobic residues in Sto-RNase HI, which have nonburied or nonhydrophobic counterparts in Tt-RNase HI, are located near the surface of the protein. Tk-RNase HII also shows a similar tendency. The results suggest that buried hydrophobic side-chains near the protein surface may contribute to slow unfolding. Unfortunately, at present, the mechanism of slow unfolding by buried hydrophobic side-chains near the protein surface remains unclear.

### Energy diagram of the stability and folding of hyperstable proteins

It has been reported that some hyperthermophilic proteins have a remarkably slow unfolding rate but a normal refolding rate in comparison with their homologous mesophilic proteins [6,8]. Tk-RNase HII unfolds extremely slowly but folds rapidly [19]. In contrast, Tt-RNase HI, which has thermostability equivalent to that of Tk-RNase HII, unfolds and folds quickly, similar to the behavior of Ec-RNase HI [19,21]. We can consider the energy diagrams of the folding process (see additional file 7). The first is for a mesophilic protein and is a two-state model. The second is for hyperstable proteins with super slow unfolding, whereas the third is for hyperstable proteins without super slow unfolding. In the second model, the increase in the equilibrium stability (ΔG) between the native and denatured states is accompanied by an increase in the unfolding barrier (N to T). In contrast, the third model includes an intermediate state in the folding process, which results in an increase in ΔG without an increase in either the folding or unfolding barriers. It has been reported that although Tt-RNase HI has an intermediate folding state, Tk-RNase HII folds according to the two-state model [19,36,60]. Therefore, Tt-RNase HI and Tk-RNase HII are represented by the third and second models, respectively. For Aa-RNase HI and Tm-RNase HI, the refolding experiments show that there is an intermediate state in the folding process (data not shown), suggesting that these proteins can be represented by the third model. When Sto-RNase HI folds according to the two-state model, k_f_(H_2_O) = 9.3 × 10 s^-1^, mf = -2.98 M^-1 ^s^-1^, and ΔG_kinetics_(H_2_O) = 73.9 kJ mol^-1 ^(unpublished data). This indicates that Sto-RNase HI is represented by the second model. On the other hand, the tetrameric protein Pf-PCP both folds and unfolds quite slowly [6]. Therefore, this protein is represented by the fourth model, in which the increase in equilibrium stability (ΔG) between the native and denatured states is accompanied by increases in both the unfolding (N to T) and folding (D to T) barriers. As a result, the unfolding barrier of the fourth model is higher than that of the second model. The unfolding rate of Pf-PCP (1.6 × 10^-15 ^s^-1^) is lower than those of Tk-RNase HII and Sto-RNase HI (6.0 × 10^-10 ^s^-1 ^and 5.7 × 10^-11 ^s^-1^) [6].

## Conclusion

In summary, we have presented the stability and unfolding kinetics of hyperstable RNases H. This is a good protein family for investigating the stability and folding mechanisms from the viewpoints of thermal adaptation and evolution because the proteins are in the monomeric form and undergo reversible unfolding. Although different mechanisms are responsible for the equilibrium stabilization of hyperstable RNases H, unfolding depends on the evolutional history of the host organisms from which the proteins originate. This has led to the use of different strategies for thermal adaptation by thermophilic archaea and bacteria.

## Methods

### Construction of plasmids

Plasmids pET800TM and pET600AA, used for overproduction of Tm-RNase HII and Aa-RNase HII, were constructed by ligating 714bp and 588bp DNA fragments, respectively, into the *Nde*I-*Sal*I sites of pET25b (Novagen). The DNA fragments were amplified by PCR using the genomic DNA of Tm and Aa as a template. The sequences of the PCR primers for Tm-RNase HII are 5'-GAAAGGAGACGG**CATATG**GGAATAGATGA-3' for the 5' primer and 5'-GCG**GTCGAC**CTTTGTGTTCGGAGATAAAG-3' for the 3' primer, and the PCR primers for Aa-RNase HII are 5'-AAC**CATATG**CTTAATTACGAATTAGAACTTTG-3' for the 5' primer and 5'-GCG**GTCGAC**CTAAAAAAGTTTTTTCTTAAGGGG-3' for the 3' primer, where the bold bases indicate the positions of the *Nde*I and *Sal*I sites. The DNA sequence of the PCR products was confirmed with a Prism 310 DNA sequencer (Perkin-Elmer).

### Overproduction and purification

*E. coli *BL21(DE3) was transformed with pET800TM and pET600AA then grown in NZCYM medium (Novagen) containing 50 mgL^-1 ^ampicillin 30 mgL^-1 ^chloramphenicol at 30°C. When the optical density at 660 nm of the culture reached 0.5, 1 mM IPTG was added to the culture medium and cultivation continued at 30°C for four hours. Cells were transformed with pET800TM, harvested by centrifugation at 8000 rpm for 10 min, suspended in 20 mM Tris-HCl (pH 8) containing 10 mM EDTA, disrupted by sonication lysis, and centrifuged at 30,000 *g *for 30 min. The supernatant was subjected to heat treatment at 75°C for 20 min and centrifuged at 30,000 *g *for 30 min. The supernatant was applied to an anion-exchange column (5 mL) of a HiTrap Q HP column (Amersham Biosciences) equilibrated with 20 mM Tris-HCl (pH 8). The protein was eluted from the column with 20 mM Tris-HCl (pH 8) containing 10 mM EDTA. The flow-through containing the protein was pooled and applied to a Hitrap Heparin and SP column (Amersham Biosciences) equilibrated with the same buffer. After the column was washed, the protein was eluted from the column with a linear gradient of NaCl from 0 to 1 M in the same buffer. The fractions containing the protein were pooled and dialyzed against 20 mM Tris-HCl (pH 8) containing 200 mM NaCl. Cells were transformed with pET600AA, harvested by centrifugation at 8000 rpm for 10 min, suspended in 20 mM Acetate-NaOH (pH 5.5) containing 10 mM EDTA, disrupted by French press lysis, and centrifuged at 30,000 *g *for 30 min. The supernatant was applied to an anion-exchange column (5 mL) of a HiTrap Q HP column equilibrated with 20 mM Acetate-NaOH (pH 5.5). The protein was eluted from the column with 20 mM Acetate-NaOH (pH 5.5) containing 10 mM EDTA. The flow-through containing the protein was pooled and applied to a Hitrap SP column equilibrated with the same buffer. After the column was washed, the protein was eluted from the column with a linear gradient of NaCl from 0 to 1 M in the same buffer. The fractions containing the protein were pooled and dialyzed against 20 mM Acetate-NaOH (pH 5.0). The fractions containing the protein were pooled and applied to a Hitrap Heparin column equilibrated with the same buffer. After the column was washed, the protein was eluted from the column with a linear gradient of NaCl from 0 to 1 M in the same buffer. The fractions containing the protein were pooled and dialyzed against 20 mM Acetate-NaOH (pH 5.0).

Sto-RNase HI was overproduced and purified as previously described [44,61]. The purity of the protein was analyzed by SDS-PAGE using a 15% polyacrylamide gel, followed with Coomassie Brilliant Blue staining.

### Protein concentration

The protein concentrations were estimated by assuming absorbances at 280 nm of 0.24 for 1 mgmL^-1 ^Tm-RNase HII and 0.89 for 1 mgmL^-1 ^Aa-RNase HII. These values were calculated using 1576 M^-1^cm^-1 ^for Tyr and 5225 M^-1^cm^-1 ^for Trp at 280 nm [62].

### CD spectra measurements

The CD spectra of Tm-RNase HII, Aa-RNase HII, and Sto-RNase HI in the absence and presence of GdnHCl were measured on a J-725 automatic spectropolarimeter (Japan Spectroscopic). The optical path length was 2 mm and the protein concentration was 0.16 mgml^-1^. The buffers we used were 20 mM Tris-HCl, 200 mM NaCl at pH 7.5 for Tm-RNase HII, 20 mM Acetate-NaOH at pH 5.0 for Aa-RNase HII, and 20 mM Glycine-NaOH at pH 3.0 for Sto-RNase HI. The measurements were done at 25°C. For the spectra of a refolded protein, the protein that was completely unfolded at a 7.0 M GdnHCl concentration was diluted with buffer for refolding, and the diluted protein solution was incubated at the selected temperature until refolding reached equilibrium. The mean residue ellipticity, θ, in units of degrees square centimeters per decimole, was calculated using an average amino acid molecular weight of 110.

### Equilibrium experiments on GdnHCl-induced unfolding

GdnHCl-induced unfolding was examined by monitoring the CD at 220 nm as described previously [19]. RNases H were incubated in GdnHCl at different concentrations and at different temperatures for unfolding. The GdnHCl-induced unfolding curves were determined, and a nonlinear least-squares analysis [63] was used to fit the data to(1)(2)

where y is the observed CD signal at a given concentration of GdnHCl, [D] is the concentration of GdnHCl, b^0^_n _is the CD signal for the native state, b^0^_u _is the CD signal for the unfolded states, a_n _is the slope of the pre-transition of the baseline, and a_u _is the slope of the posttransition of the baseline. ΔG(H_2_O) is the Gibbs energy change (ΔG) of the unfolding in the absence of GdnHCl, m is the slope of the linear correlation between ΔG and the GdnHCl concentration [D], and C_m _is the GdnHCl concentration at the midpoint of the curve. The raw experiment data were directly fitted to Eq. (1) using SigmaPlot (Jandel Scientific). The equilibrium experiments were conducted in 20 mM Tris-HCl, 200 mM NaCl at pH 7.5 for Tm-RNase HII, 20 mM Acetate-NaOH at pH 5.0 for Aa-RNase HII, and 20 mM Glycine-NaOH at pH 3.0 for Sto-RNase HI. To use Tris-HCl buffer at different temperatures, the pH was adjusted at 25°C so that the final pH would be 7.5 at the desired temperature [64]. The protein concentration when the CD measurements were taken was 0.16 mgmL^-1^.

### Stability curves

ΔG(H_2_O) was examined at various temperatures to determine the stability curves for Tm-RNase HII, Aa-RNase HII, and Sto-RNase HI. The stability curves obtained were fitted to the Gibbs-Helmholtz equation, Eq. (3):(3)

where ΔH(T_o_) and ΔS(T_o_) are the enthalpy and entropy of unfolding at the reference temperature T_o_, and ΔC_p _is the difference in heat capacity between the native and unfolded states.

### Heat-induced unfolding experiments

Heat-induced unfolding was examined by monitoring the CD at 220 nm. CD measurements were carried out on a J-725 automatic spectropolarimeter as described previously [65]. The optical path length was 2 mm. The thermal denaturation curves of these proteins were measured in the presence of various concentrations of GdnHCl to determine the T_m _values of Tm-RNase HII and Aa-RNase HII in the absence of GdnHCl, where thermal denaturation of these proteins is not reversible. The buffer used was 20 mM Tris-HCl, 200 mM NaCl at pH 7.5 in the presence of 1.0 to 2.0 M GdnHCl for Tm-RNase HII and 20 mM Acetate-NaOH at pH 5.0 in the presence of 1.2 to 2.0 M GdnHCl for Aa-RNase HII. The thermal denaturation of these proteins was reversible under these conditions. The protein concentration was 0.16 mgml^-1^. All experiments were carried out at a scan rate of 1 °Cmin^-1^. A nonlinear least-squares analysis [66] was used to fit the data to(4)

where y is the observed CD signal at a given temperature [T], b_n _is the CD signal for the native state, b_u _is the CD signal for the unfolded states, a_n _is the slope of the pretransition of the baseline, a_u _is the slope of the posttransition of the baseline, ΔH_m _is the enthalpy of unfolding at the transition midpoint temperature (T_m_), T is the temperature, and R is the gas constant. Curve fitting was performed using SigmaPlot. Because the T_m _values of these proteins increased in proportion to a decrease in GdnHCl concentration, the T_m _values in the absence of GdnHCl were estimated by linear extrapolation [46].

### Kinetic experiments on GdnHCl-induced unfolding

The unfolding reactions were followed by CD spectra measurement at 220 nm, using 2 mm and 1 cm path length cuvettes as described previously [19]. CD measurements were conducted using a J-725 automatic spectropolarimeter. The unfolding reactions of proteins were induced by a concentration jump in GdnHCl, with various differing concentrations. The protein solution was stirred using a spinning mixer with a magnetic stirrer in a cuvette with a 1 cm path length for the unfolding, and the CD was recorded at 220 nm as a function of time. The dead time of this method was 2 s. The protein solution was stirred manually during use of a cuvette with a 2 mm path length. The dead time of this method was 10 s. The kinetic experiments were performed in 20 mM Tris-HCl, 200 mM NaCl at pH 7.5 for Tm-RNase HII, 20 mM Acetate-NaOH at pH 5.0 for Aa-RNase HII, and 20 mM Glycine-NaOH at pH 3.0 for Sto-RNase HI. The protein concentrations when CD measurements were taken were 0.0338 to 0.169 mgmL^-1^. The kinetic data were analyzed using Eq. (5).(5)

Here, A(t) is the value of the CD signal at a given time t, A(∞) is the value when no further change is observed, k is the apparent rate constant, and A is the amplitude. The GdnHCl concentration dependence of the logarithms of the apparent rate constant (k_app_) for unfolding was also examined. The rate constants for unfolding in the absence of GdnHCl (k_u_(H_2_O)) were calculated by fitting to Eq. (6):(6)

where [D] is the concentration of GdnHCl and m_u _represents the slopes of the linear correlations of ln k_u _with the GdnHCl concentration.

## Abbreviations

Aa: *Aquifex aeolicus*; CD: circular dichroism; Csp: cold shock protein; ΔG(H_2_O): Gibbs energy change (ΔG) of the unfolding in water; Ec: *Escherichia coli*; GdnHCl: guanidine hydrochloride; PCP: pyrrolidone carboxyl peptidase; Pf: *Pyrococcus furiosus*; RNase: ribonuclease; S16: ribosomal protein S16; Sto: *Sulfolobus tokodaii*; Tk: *Thermococcus kodakaraensis*; Tm: *Thermotoga maritima*; T_m_: thermal denaturation temperature; Tt: *Thermus thermophilus*

## Authors' contributions

KT conceived and supervised the study. JO and TO conducted all experimental work. AM, TT, DJY and HC helped data analyses. YK and SK helped in interpretation of data and discussion of results. All authors read and approved the manuscript.

## Supplementary Material

Additional file 1 **Crystal structures of (A) Ec-RNase HI (PDB ID: **2RN2)**, (B) Tt-RNase HI (1RIL), (C) Sto-RNase HI (2EHG), (D) Tk-RNase HII (1IO2), and (E) Tm-RNase HII (2ETJ)**. Click here for file

Additional file 2 **Far-UV CD spectra of RNases H at 25°C**. Click here for file

Additional file 3 **GdnHCl-induced denaturation curves of RNases H at 20°C**. Click here for file

Additional file 4 **Pie diagrams representing the fraction of hydrophobic, polar, and charged residues in the interior of the tertiary structure of hyperstable RNases H**. (A) RNase HII from *Pyrococcus furiosus*. (B) RNase HII from *Achaeoglobus fulgidus*. Accessible surface and buried area of RNases H. Click here for file

Additional file 5 **Phylogenetic tree of thermostable RNases H on the basis of the amino acid sequences**. Click here for file

Additional file 6 **Accessible surface and buried area of RNases H**. Click here for file

Additional file 7 **Schematic representations of the energy diagrams of the protein folding process**. Click here for file
