# Supplementary material for: Evolution and thermodynamics of the slow unfolding of hyperstable monomeric proteins
Source: BMC Evol Biol. 2010 Jul 9;10:207. doi: 10.1186/1471-2148-10-207 (PMC2927913; doi:10.1186/1471-2148-10-207)

Additional file 1. Crystal structures of (A) Ec-RNase HI (PDB ID: 2RN2), (B) Tt-RNase HI (1RIL), (C) Sto-RNase HI (2EHG), (D) Tk-RNase HII (1IO2), and (E) Tm-RNase HII (2ETJ). The figures were created by PyMOL [S1].

S1. DeLano WL PyMOL User's Guide. DeLano Scientific San Carlos, California. 2004

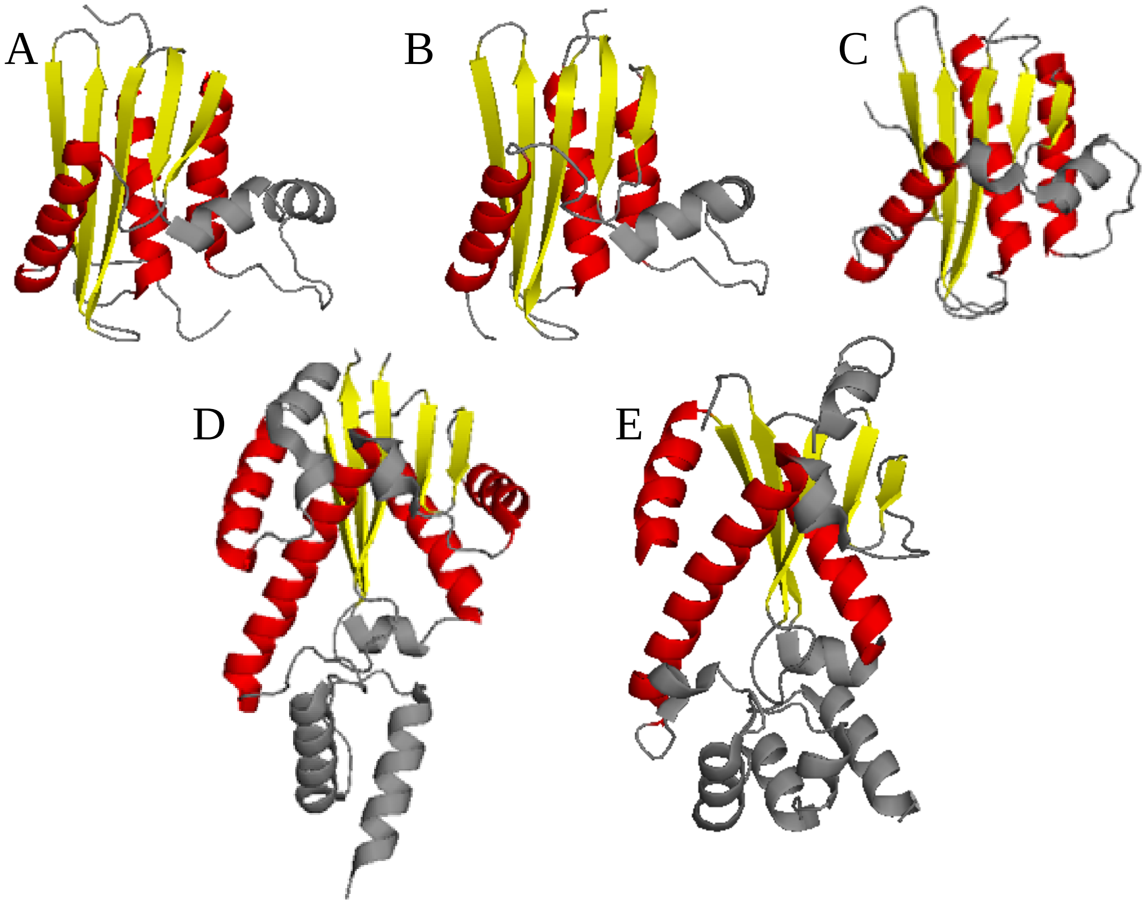

Supplement: Additional file 1 — Crystal structures of (A) Ec-RNase HI (PDB ID: 2RN2), (B) Tt-RNase HI (1RIL), (C) Sto-RNase HI (2EHG), (D) Tk-RNase HII (1IO2), and (E) Tm-RNase HII (2ETJ). [file 1471-2148-10-207-S1.PDF]
