# Supplementary material for: Evolution and thermodynamics of the slow unfolding of hyperstable monomeric proteins
Source: BMC Evol Biol. 2010 Jul 9;10:207. doi: 10.1186/1471-2148-10-207 (PMC2927913; doi:10.1186/1471-2148-10-207)
Supplement: Additional file 2 — Far-UV CD spectra of RNases H at 25°C. [file 1471-2148-10-207-S2.PDF]

Additional file 2. Far-UV CD spectra of RNases H at 25°C. (A) The spectra of Tm-RNase HII were measured at pH 7.5 in the absence (thick line) and presence (thin line) of 4.0M GdnHCl. The dashed line represents the spectrum of the refolded protein in the presence of 1.5M GdnHCl. (B) The spectra of Aa-RNase HII were measured at pH 5.0 in the absence (thick line) and presence (thin line) of 4.0M GdnHCl. The dashed line represents the spectrum of the refolded protein in the presence of 1.5M GdnHCl. (C) The spectra of Sto-RNase HI were measured at pH 3.0 in the absence (thick line) and presence (thin line) of 6.4M GdnHCl. The dashed line represents the spectrum of the refolded protein in the presence of 1.0M GdnHCl.

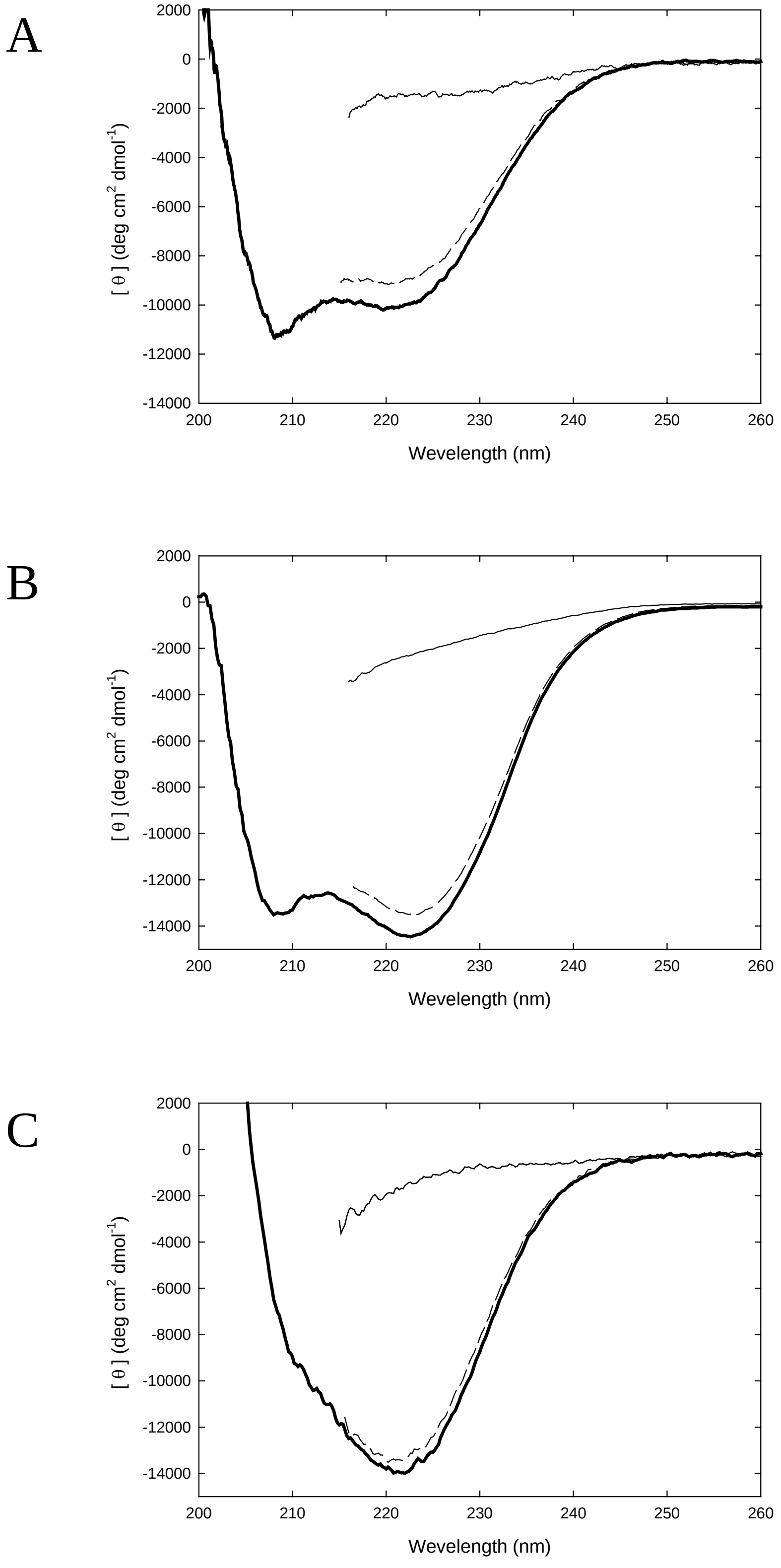
A
B
C
[θ] (deg cm^2 dmol^{-1})
Wavelength (nm)
2000
0
-2000
-4000
-6000
-8000
-10000
-12000
-14000
200
210
220
230
240
250
260
