# Supplementary material for: Evolution and thermodynamics of the slow unfolding of hyperstable monomeric proteins
Source: BMC Evol Biol. 2010 Jul 9;10:207. doi: 10.1186/1471-2148-10-207 (PMC2927913; doi:10.1186/1471-2148-10-207)

Additional file 3. GdnHCl-induced denaturation curves of RNases H at 20°C. Open circles represent the GdnHCl-induced denaturation curves of Tm-RNase HII at pH 7.5; open triangles, those of Aa-RNase HII at pH 5.0; and closed squares, those of Sto-RNase HI at pH 3.0. The lines represent the fit of Eq. (1).

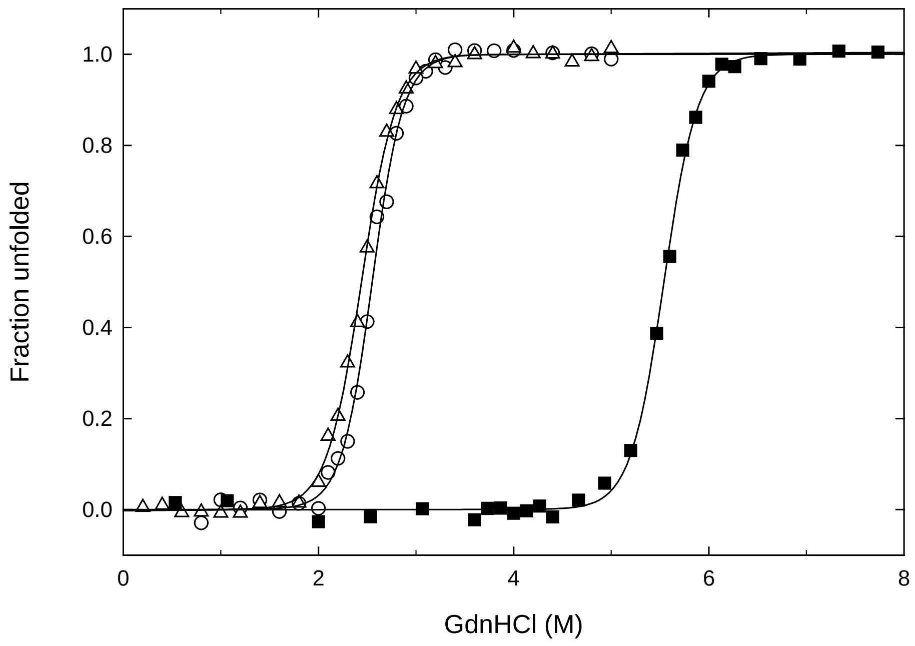

Supplement: Additional file 3 — GdnHCl-induced denaturation curves of RNases H at 20°C. [file 1471-2148-10-207-S3.PDF]
