# Supplementary material for: Evolution and thermodynamics of the slow unfolding of hyperstable monomeric proteins
Source: BMC Evol Biol. 2010 Jul 9;10:207. doi: 10.1186/1471-2148-10-207 (PMC2927913; doi:10.1186/1471-2148-10-207)

Additional file 4. Pie diagrams representing the fraction of hydrophobic, polar, and charged residues in the interior of the tertiary structure of hyperstable RNases H. (A) RNase HII from *Pyrococcus furiosus*. (B) RNase HII from *Achaeoglobus fulgidus*. Blue denotes positively charged residues (Arg and Lys). Red denotes negatively charged residues (Asp and Glu). Green denotes polar residues (Asn, Gln, Ser, and Thr). Yellow denotes hydrophobic residues (Ile, Leu, Met, Phe, Trp, Tyr, and Val). White denotes other residues (Ala, Cys, Gly, His, and Pro). Amino-acid residues with relative solvent accessibility greater than 25% were regarded as residues exposed to solvent [S2].

S2. You DJ, Fukuchi S, Nishikawa K, Koga Y, Takano K, Kanaya S: **Protein thermostabilization requires a fine-tuned placement of surface-charged residues.** *J Biochem* 2007, **142:**507-516.

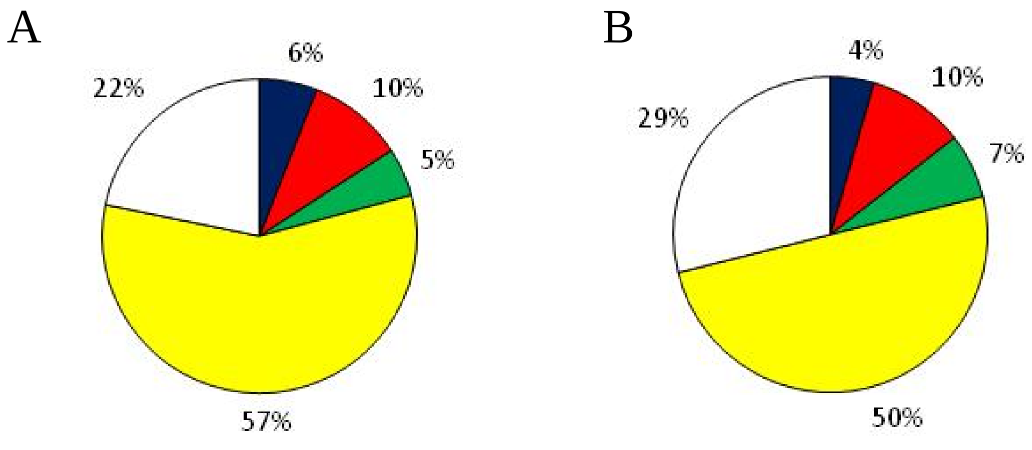

Supplement: Additional file 4 — Pie diagrams representing the fraction of hydrophobic, polar, and charged residues in the interior of the tertiary structure of hyperstable RNases H. (A) RNase HII from Pyrococcus furiosus. (B) RNase HII from Achaeoglobus fulgidus. Accessible surface and buried area of RNases H. [file 1471-2148-10-207-S4.PDF]
