# Supplementary material for: Evolution and thermodynamics of the slow unfolding of hyperstable monomeric proteins
Source: BMC Evol Biol. 2010 Jul 9;10:207. doi: 10.1186/1471-2148-10-207 (PMC2927913; doi:10.1186/1471-2148-10-207)
Supplement: Additional file 5 — Phylogenetic tree of thermostable RNases H on the basis of the amino acid sequences. [file 1471-2148-10-207-S5.PDF]

Additional file 5. Phylogenetic tree of thermostable RNases H on the basis of the amino acid sequences. Alignments and trees were calculated using the alignment programs ClustalW with the default parameters [S3]. The resulting trees were plotted using Treeview [S4]. Optimal growth temperature of each organism is shown in parenthesis. RNases HII (accession number) are *Aeropyrum pernix* (NP_147280), *Archaeoglobus fulgidus* (O29634), *Methanocaldococcus jannaschii* (NP_247101), *Methanopyrus kandleri* (NP_613473), *Methanothermobacter thermautotrophicus* (NP_276158), *Picrophilus torridus* (YP_024041), *Pyrococcus furiosus* (NP_579510), *Pyrococcus horikoshii* (NP_143500), *Sulfolobus solfataricus* (NP_343742), *Sulfolobus tokodaii* (NP_376405), *Thermococcus kodakarenisis* (YP_183218), *Thermoplasma acidophilum* (NP_394911), *Thermoplasma volcanium* (NP_110652), *Aquifex aeolicus* (NP_214337), *Escherichia coli* (YP_001729139), *Geobacillus kaustophilus* (YP_147058), *Thermoanaerobactr tengcongensis* (NP_623069), *Thermobifida fusca* (YP_290201), *Thermosynechococcus elongatus* (NP_681452), *Thermotoga maritima* (NP_228723), *Thermus thermophilus* (YP_143464). RNases HI (accession number) are *Sulfolobus acidocaldarius* (YP_256320), *Sulfolobus tokodaii* (NP_376653), *Escherichia coli* (YP_001729165), *Thermosynechococcus elongates* (NP_681090), *Thermoanaerobacter tengcongensis* (NP_622980), *Thermus thermophilus* (YP_144822).

S3. Thompson JD, Higgins DG, Gibson TJ: **Clustal W: Improving the sensitivity of progressive multiple sequence alignment through sequence weighting, position-specific gap penalties and weight matrix choice.** *Nucl Acids Res* 1994, **22:** 4673-4680.

S4. Page RD: **Treeview: An application to display phylogenetic trees on personal computers.** *Comput Appl Biosci* 1996, **12:** 357-358.

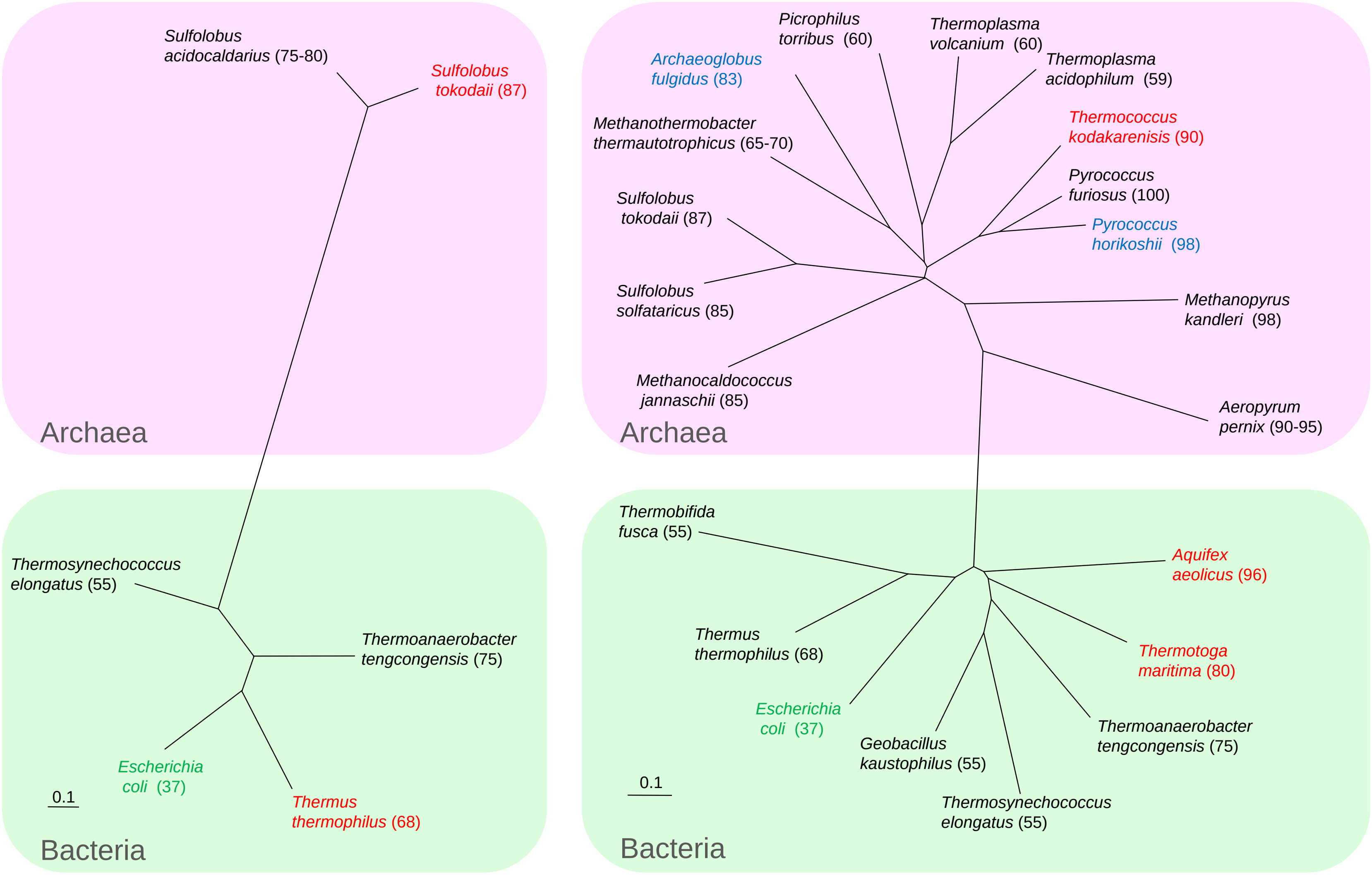

RNase HI

RNase HII
