# Supplementary material for: Evolution and thermodynamics of the slow unfolding of hyperstable monomeric proteins
Source: BMC Evol Biol. 2010 Jul 9;10:207. doi: 10.1186/1471-2148-10-207 (PMC2927913; doi:10.1186/1471-2148-10-207)
Supplement: Additional file 6 — Accessible surface and buried area of RNases H. [file 1471-2148-10-207-S6.PDF]

Additional file 6. Accessible surface and buried area of RNases H.

	Tt-RNase HI	Sto-RNase HI	Tk-RNase HII
Accessible surface area of native state (Å^2)[a]			
All	8172	8384	10829
Non-polar	4545	4642	5912
Polar	3627	3742	4917
Accessible surface area of denatured state (Å^2)[ab]			
All	23441	23061	32937
Non-polar	14102	14524	20169
Polar	9339	8537	12768
Buried area in the interior of a protein (Å^2)			
All	15269	14677	22108
Non-polar	9559	9882	14258
Polar	5712	4795	7851
Fraction of the buried area of non-polar atoms (%)	62.6	67.3	64.5

[a] Accessible surface area is calculated by POPS [S5].

[b] The denatured structure is created by PyMOL [S1] as a completely extended conformation.

S1. DeLano WL PyMOL User’s Guide. DeLano Scientific San Carlos, California. 2004

S5. Cavallo L, Kleinjung J, Fraternali F: **POPS: A fast algorithm for solvent accessible surface areas at atomic and residue level.** *Nucleic Acids Res* 2003, **31**:3364-3366.
