# Supplementary material for: Evolution and thermodynamics of the slow unfolding of hyperstable monomeric proteins
Source: BMC Evol Biol. 2010 Jul 9;10:207. doi: 10.1186/1471-2148-10-207 (PMC2927913; doi:10.1186/1471-2148-10-207)

**Additional file 7**. Schematic representations of the energy diagrams of the protein folding process. (A) Mesophilic protein with a two-state model. (B) Hyperstable protein with super-slow unfolding. (C) Hyperstable protein without super-slow unfolding. (D) Hyperstable protein with super-slow unfolding and refolding. N, T, D, and I represent the native, transition, denatured, and intermediate states.

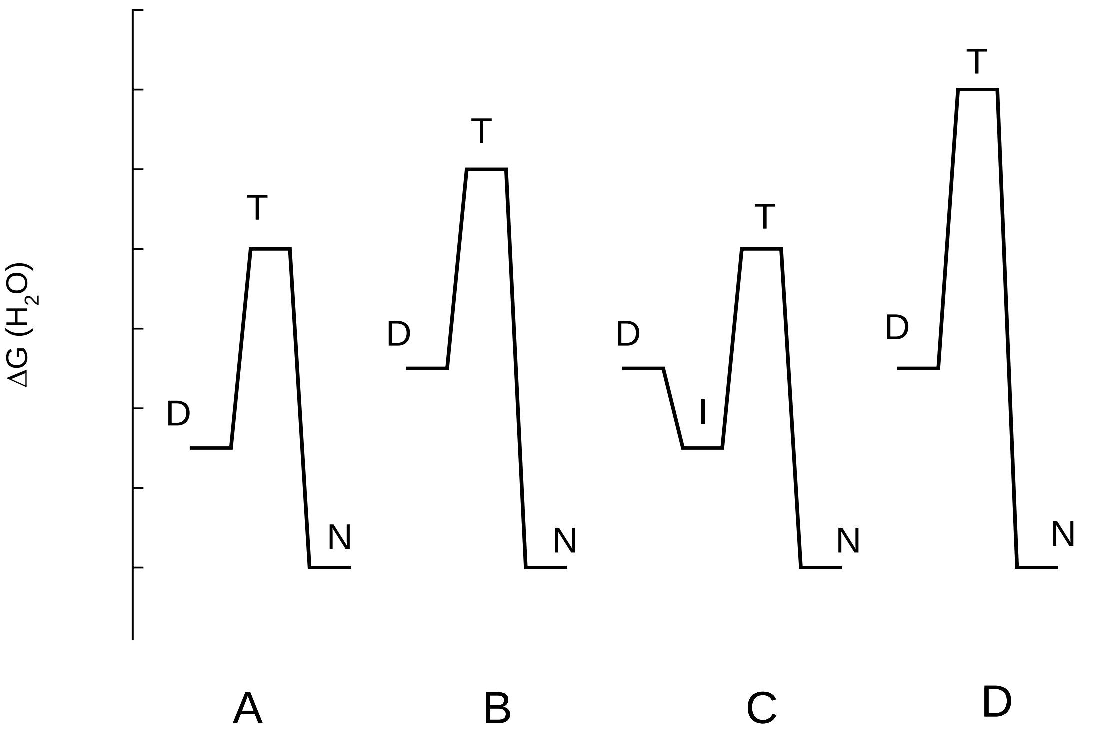

Supplement: Additional file 7 — Schematic representations of the energy diagrams of the protein folding process. [file 1471-2148-10-207-S7.PDF]
